# Association between the hemoglobin albumin lymphocyte and platelet score and chronic kidney disease: Insights from patient data and animal models

**DOI:** 10.1371/journal.pone.0353085

**Published:** 2026-10-14

**Authors:** Wenyu Zhang, Yashi Wang, Wenfeng Ye, Yiman Wang, Xuejing Chen, Bingwu Zhao, Xueqin Zhang, Zhiqiang Chen

**Affiliations:** 1 Hebei University of Chinese Medicine, Shijiazhuang, Hebei, China; 2 The First Affiliated Hospital of Hebei University of Chinese Medicine, Shijiazhuang, Hebei, China; The University of the West Indies, JAMAICA

## Abstract

**Objectives:**

The hemoglobin, albumin, lymphocytes and platelets (HALP) score, a novel nutritional and inflammatory biomarker, has been used in various chronic disease studies. However, the relationship between the HALP score and chronic kidney disease (CKD) remains poorly elucidated. This study aimed to explore the association between the HALP score and CKD.

**Methods:**

Our analysis encompassed 25,181 adult participants drawn from NHANES cycles spanning 2009–2018. Weighted multivariable logistic regression and generalized additive models (GAMs) were employed to evaluate the independent associations between the HALP score and three outcomes: CKD, albuminuria, and low-estimated glomerular filtration rate (eGFR). CKD was defined as albuminuria (urinary albumin-to-creatinine ratio ≥ 30 mg/g) or low-eGFR (eGFR < 60 mL/min/1.73m^2^). Threshold effects were examined using two-piecewise linear regression. Subgroup and sensitivity analyses were performed to assess robustness. Receiver operating characteristic (ROC) curve analyses were applied to compare the discriminative capacity of the HALP score with the prognostic nutritional index (PNI), systemic immune-inflammation index (SII), lymphocyte-to-monocyte ratio (LMR), and platelet-to-lymphocyte ratio (PLR). A 5/6 nephrectomy rat model of CKD was established to evaluate the HALP values and their components.

**Results:**

After adjustment for multiple confounders, higher HALP scores were inversely associated with prevalent CKD (OR = 0.97, 95% CI: 0.94–0.99) and albuminuria (OR = 0.97, 95% CI: 0.93–0.99). However, the association between the HALP score and low-eGFR was no longer statistically significant in Model 3. Non-linear analyses revealed a threshold pattern, with higher HALP scores inversely associated with prevalent CKD up to an inflection point of 52.43 (OR = 0.97, 95% CI: 0.95–0.99), beyond which no significant association was observed. For albuminuria, the corresponding inflection point was 49.82. Subgroup and interaction analyses indicated no meaningful effect modification by age, sex, BMI, hypertension, or diabetes. Sensitivity analyses confirmed the robustness of the results. ROC analysis demonstrated that the HALP score had modestly higher AUCs for CKD and albuminuria compared with PNI, SII, LMR, and PLR. In the animal experiment, CKD rats exhibited significantly lower calculated HALP values, which were inversely correlated with serum creatinine (Scr), blood urea nitrogen (BUN), and urinary albumin-to-creatinine ratio (UACR), further supporting the biological plausibility of the HALP–CKD association.

**Conclusion:**

In this cross-sectional study, lower HALP scores are independently associated with a higher prevalence of CKD and albuminuria. As an affordable and readily measurable composite index, a low HALP score may represent a complementary marker of prevalent CKD, while its clinical utility requires prospective validation.

## Introduction

Chronic kidney disease (CKD) represents a critical public health concern globally, impacting several hundred million people and serving as a significant driver of disease burden and mortality worldwide [1]. A critical clinical challenge is that CKD is frequently asymptomatic in its early stages, which delays timely diagnosis. By the time clinical manifestations emerge, significant and often irreversible kidney injury has already occurred [2]. Therefore, early detection, prompt intervention, and effective management are essential for reducing the disease burden, highlighting the importance of identifying reliable risk markers for CKD progression. Accumulating evidence suggests that CKD progression is closely linked to persistent subclinical inflammation, a key mediator of kidney injury and renal function deterioration [3]. In addition to inflammation, malnutrition is also a recognized contributor to CKD development [4]. As the disease progresses, malnutrition tends to worsen, further adversely affecting patient outcomes. A bidirectional “inflammation-malnutrition” interaction has thus been proposed, suggesting that composite indicators integrating both dimensions may better capture CKD risk than single markers [5]. Indeed, growing evidence has demonstrated that several nutrition- and inflammation-related markers can serve as reliable indicators of CKD, including the systemic inflammation response index (SIRI) [6] and the advanced lung cancer inflammation index (ALI) [7]. Initially formulated as a prognostic indicator for individuals with gastric malignancies, the HALP score simultaneously captures both the body’s inflammatory state and its nutritional condition concurrently [8]. Subsequently, its prognostic utility has been validated in a range of kidney disorders, such as diabetic nephropathy (DN) [9], immunoglobulin A nephropathy (IgAN) [10], lupus nephritis [11], and for predicting clinical outcomes in dialysis patients [12,13]. However, existing research on the HALP score in renal disease has predominantly focused on specific subtypes such as DN and IgAN, as well as dialysis populations, while evidence from general-population cohorts remains scarce. Moreover, complementary experimental evidence regarding the CKD-related biological features reflected by HALP remains limited. To address this gap, we utilized NHANES data to examine the associations between the HALP score and CKD, albuminuria, and low-eGFR. In parallel, a 5/6 nephrectomy rat model was established to provide experimental context for the CKD-related biological features represented by the HALP score. If supported by prospective validation, the HALP score, calculated from routinely available laboratory parameters, could be explored as a simple adjunct to help identify individuals for further renal assessment.

## Materials and methods

### Data sources

Data analyzed in this study were obtained from NHANES, a continuous, nationally representative health survey administered by the NCHS that employs a complex multistage probability sampling framework. Ethical approval and written informed consent had already been secured under the original NHANES protocol; no further review was therefore required for this secondary analysis of de-identified public-use files.

### Data collection and measures

Data from NHANES 2009–2018 were utilized for this analysis, including 49,693 participants. Exclusion criteria comprised participants younger than 20 years (n = 20,858), those who were pregnant (n = 315), and individuals with missing serum creatinine (Scr) or urinary albumin-to-creatinine ratio (ACR) data (n = 3,201). Subjects with insufficient information for HALP score computation (n = 83) were also omitted, resulting in 25,236 eligible participants. To address the potential confounding effects of renal replacement therapy, participants undergoing hemodialysis or peritoneal dialysis (n = 55) were further excluded. Consequently, the final analytic cohort included 25,181 participants (Fig 1).

**Fig 1:**
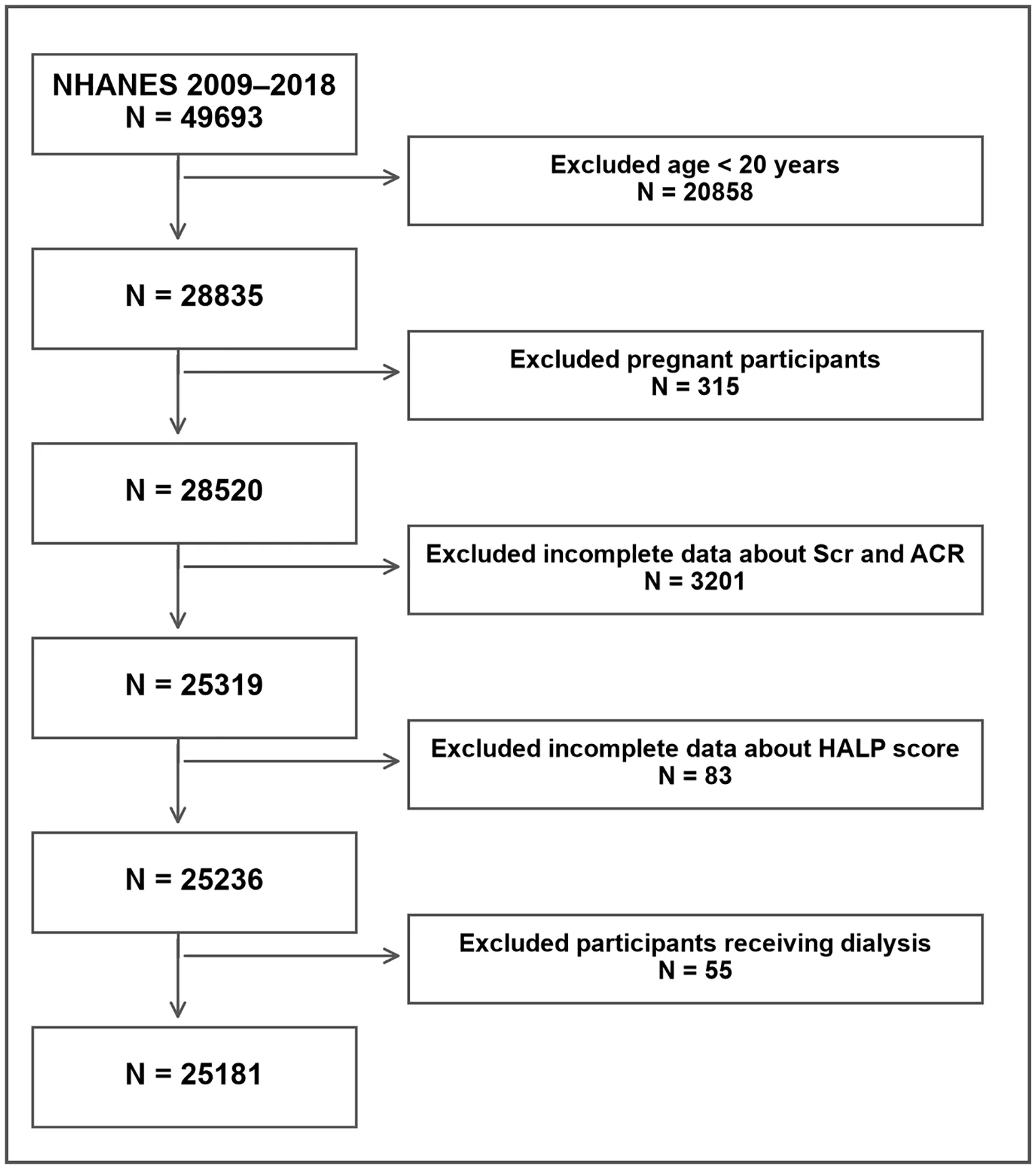
Flowchart of participants selection. NHANES = National Health and Nutrition Examination Survey, Scr = serum creatinine, ACR = urine albumin-to-creatinine ratio, HALP score = hemoglobin, albumin, lymphocyte, and platelet score.

### Assessment of HALP score

The HALP score was treated as the primary exposure variable and calculated as follows: HALP score = [hemoglobin (g/L) × serum albumin (g/L) × lymphocyte count (10^9^/L)] / platelet count (10^9^/L). Hematological parameters were determined through an automated blood cell analyzer (Beckman Coulter), while quantification of serum albumin relied on the bromocresol purple binding assay.

### Assessment of CKD

CKD was determined based on albuminuria or a low-estimated glomerular filtration rate (eGFR). An eGFR < 60 mL/min/1.73 m^2^ was considered low, with eGFR calculated using the 2009 Chronic Kidney Disease Epidemiology Collaboration (CKD-EPI) equation. Albuminuria corresponded to an ACR of ≥ 30 mg/g. Because CKD was assessed at a single NHANES visit, KDIGO chronicity (≥3 months) could not be confirmed. For analysis, CKD, albuminuria, and low-eGFR were examined separately as outcomes.

### Covariates

Demographic variables included age (< 60 years or ≥ 60 years), gender (male or female), race (Mexican American, Other Hispanic, Non-Hispanic White, Non-Hispanic Black, and Other), poverty-to-income ratio (PIR; < 1, 1–4, or > 4), education levels (high school or less and more than high school), and marital status (married, widowed, divorced, separated, never married, or living with a partner). Physical and laboratory parameters assessed were body mass index (BMI; < 25, 25–30, or ≥ 30 kg/m^2^), eGFR, ACR, blood urea nitrogen (BUN), Scr, serum uric acid (SUA), hemoglobin (HGB), lymphocytes (LYM), platelets (PLT), serum albumin (ALB), alanine transaminase (ALT), aspartate transaminase (AST), low-density lipoprotein cholesterol (LDL-C), high-density lipoprotein cholesterol (HDL-C), total cholesterol (TC), and triglycerides (TG). Health-related behaviors and comorbidities were also incorporated. Smoking status was dichotomized as yes/no, defined by lifetime cigarette consumption ≥ 100 sticks. Drinking status was categorized as yes/no, based on annual alcoholic beverage intake exceeding 12 servings. Hypertension was defined as mean systolic blood pressure ≥ 140 mmHg, mean diastolic blood pressure ≥ 90 mmHg, self-reported hypertension, or current use of antihypertensive medications. Diabetes mellitus (DM) was identified by glycohemoglobin ≥ 6.5%, fasting glucose ≥ 7.0 mmol/L, a confirmed medical diagnosis of DM, or ongoing use of DM medications or insulin therapy. Participants who ever reported having congestive heart failure, coronary heart disease, angina pectoris or heart attack were diagnosed as having cardiovascular disease (CVD). Participants with TC ≥ 5.2 mmol/L, TG ≥ 1.7 mmol/L, LDL-C ≥ 3.4 mmol/L, HDL-C ≤ 1.0 mmol/L, self-reported hypercholesterolemia, or on lipid-lowering therapy were identified as having dyslipidemia.

### Animal experimental design

#### Experimental animals.

All experimental procedures involving animals were approved by the Animal Ethics Committee of Hebei Provincial Hospital of Traditional Chinese Medicine (IACUC-HPHCM-2026015). Male Sprague-Dawley (SD) rats (200 ± 20 g body weight) were procured from Liaoning Changsheng Biotechnology Co., Ltd. Animals were maintained in specific pathogen-free (SPF) facilities at the Experimental Animal Center under controlled environmental conditions (22 ± 2 °C, 50 ± 10% humidity, 12-h photoperiod) with unrestricted access to standard diet and drinking water. Experiments were conducted in line with local animal welfare standards and the 3R principles to alleviate animal discomfort. All animal procedures were conducted by investigators certified in laboratory animal handling.

#### CKD model establishment.

After a one-week acclimatization period, rats were randomly allocated to the control (CON) group or the CKD model group (n = 10 per group). The CKD model was established using the two-step 5/6 nephrectomy procedure (Platt’s method) with minor modifications [14]. Briefly, rats were anesthetized via intraperitoneal injection of 1% pentobarbital sodium (50 mg/kg), with adequate anesthetic depth confirmed by the absence of the pedal withdrawal reflex prior to surgery. Following routine skin preparation and disinfection, a longitudinal incision was made at the left costovertebral angle to expose the left kidney. Approximately one-third of both the upper and lower poles of the renal cortex were resected separately, and hemostasis was achieved using gelatin sponges. The kidney was repositioned, and the incision was closed in layers. One week later, a total right nephrectomy was performed: the right renal pedicle was carefully ligated to prevent bleeding, and the kidney was excised intact before the abdominal cavity was closed in layers. Postoperative analgesia was provided by subcutaneous buprenorphine (0.05 mg/kg) at 12-h intervals for 48 h after each surgery, and animals were warmed on a heating pad until they had fully recovered from anesthesia.

Two weeks after the second surgery, successful CKD model establishment was confirmed by elevated serum Scr and BUN levels and characteristic renal histopathological changes. The total experimental duration was 4 weeks from the initial surgery (1 week between the two surgeries plus 2 weeks of post-modeling observation/treatment).

#### Animal monitoring and humane endpoints.

Throughout the entire study, the health and behavior of every animal were assessed no less than once per day, and body weight was documented on two occasions each week. Observation indicators comprised body weight, intake of food and water, body posture, level of activity, fur appearance, breathing pattern, and healing of the surgical incision. The pre-established criteria mandating immediate euthanasia were as follows: (1) a reduction in body weight of more than 20% relative to baseline; (2) failure to consume food or water; (3) marked respiratory difficulty; (4) a sustained moribund condition or lack of response to external stimuli; (5) manifestations of intense or refractory pain or distress (such as hunched posture, piloerection, or self-mutilation); or (6) wound rupture or severe infection that did not respond to veterinary treatment.

Of the 20 rats enrolled, all reached the scheduled study endpoint and were euthanized according to the planned protocol. No unexpected deaths occurred during the experiment, and none of the animals met the pre-established criteria that would have warranted earlier euthanasia.

#### Euthanasia and sample collection.

Following the 2-week post-modeling treatment phase, all rats were fasted overnight before being euthanized with isoflurane. In brief, anesthetic induction was achieved by exposing animals to 3–5% isoflurane in oxygen within an induction chamber, after which the concentration was lowered to 1.5–2.5% for maintenance until nociceptive reflexes were entirely abolished, as verified by the disappearance of both pedal withdrawal and corneal responses. Whole blood was subsequently obtained through abdominal aortic puncture under aseptic conditions, and serum was separated by centrifugation. Throughout this procedure, isoflurane delivery was sustained until respiration and cardiac activity had completely ceased, thereby confirming death. Residual kidneys were then aseptically excised and decapsulated, and a portion of each kidney was immersed in 4% paraformaldehyde for 48 h for subsequent histological evaluation.

#### Biochemical analyses.

Serum concentrations of BUN, Scr, and ALB were measured with commercial assay kits (Raydu Biotech, Wuhan, China) according to the manufacturer’s instructions, using the urease–glutamate dehydrogenase method (Cat. No. S036), an enzymatic assay (Cat. No. S03076), and the bromocresol green method (Cat. No. S03043), respectively. Urinary albumin and creatinine were determined in matched urine samples with the Rat Microalbuminuria ELISA Kit (Cat. No. E-EL-R0025) and the Creatinine Colorimetric Assay Kit (Cat. No. E-BC-K188-M; both from Elabscience, Wuhan, China), and the urinary albumin-to-creatinine ratio (UACR) was calculated accordingly. Hemoglobin (Hb), lymphocyte (LYM), and platelet (PLT) counts were determined on a Mindray BC-2800Vet veterinary hematology analyzer (Mindray, Shenzhen, China). HALP values were subsequently calculated from Hb, ALB, LYM, and PLT according to the formula described in Section 2.3. The human-derived HALP cutoff was not applied to the animal data.

#### Histopathological analysis.

Continuous 4-μm sections underwent H&E and Masson trichrome staining for assessment of glomerular morphology, tubular injury, and interstitial fibrosis. Histopathological assessment was performed by an investigator blinded to group allocation.

### Statistical analysis

#### NHANES analysis.

All analyses incorporated NHANES survey design and sampling weights to ensure national representativeness, in accordance with NHANES analytical guidelines. Data for continuous variables are presented as mean ± SD, and categorical data are summarized using proportions. To assess differences in baseline profiles across quartiles of the HALP score, weighted linear regression was applied to continuous measures, while weighted chi-square testing was employed for categorical ones.

The relationships of the HALP score with CKD, albuminuria, and low-eGFR were investigated through weighted multivariable logistic regression, implemented across three sequential models. The crude model (Model 1) included no adjustments; Model 2 incorporated demographic factors including age, gender, and race; Model 3 additionally controlled for marital status, educational level, PIR, BMI, smoking and drinking, AST, ALT, hypertension, DM, CVD, and dyslipidemia.

To explore potential non-linear associations, smooth curve fitting and generalized additive models (GAMs) were applied, followed by a two-piecewise linear regression model to identify threshold effects. The inflection point was identified by maximum likelihood estimation among observed HALP scores within the 5th–95th percentiles, with no prespecified cut points imposed; its 95% CI was obtained by profile likelihood. Stratified analyses by age, gender, BMI, hypertension, and DM were performed, and interactions were assessed using likelihood ratio tests. For sensitivity analyses, Model 3 was rerun after excluding participants with HALP scores in the top and bottom 5% of the distribution. ROC curve analysis was used to compare the discriminative ability of the HALP score, PNI, PLR, LMR, and SII. Pairwise AUC comparisons were performed using DeLong’s test. Missing covariate data were handled using multiple imputation. Data processing relied on R software (version 4.1.3) and EmpowerStats, with significance defined at a two-sided threshold of *P* < 0.05.

#### Animal experiment analysis.

For animal experiments, statistical analyses were performed using SPSS version 26.0. Data are expressed as mean ± standard error of the mean (SEM). Independent-samples t-tests were used to compare renal function indicators and HALP values between the CON and CKD model groups. Pearson correlation analysis was performed to examine the associations between HALP values and serum BUN, Scr, and UACR concentrations in all rats.

## Results

### Baseline characteristics

A total of 25,181 individuals participated, with a mean age of 50.79 ± 14.60 years, comprising 48.95% males and 51.05% females. The overall prevalence of CKD was 17.10%. Age, gender, marital status, BMI, CKD, eGFR, ACR, BUN, Scr, SUA, HGB, LYM, PLT, ALB, ALT, AST, TG, and smoking status differed significantly across HALP score quartiles (all *P* < 0.05) (Table 1).

**Table 1:** Baseline characteristics according to HALP score quartiles.

Characteristics	Total	Quartile 1 <40.25	Quartile 2 40.25–52.47	Quartile 3 52.47–68.43	Quartile 4 ≥68.43	*P* value
*N*	25181	6296	6295	6295	6295	
Age group (%)						<0.001
<60	16778 (66.63)	3723 (59.13)	4089 (64.96)	4360 (69.26)	4606 (73.17)	
≥60	8403 (33.37)	2573 (40.87)	2206 (35.04)	1935 (30.74)	1689 (26.83)	
Gender (%)						<0.001
Male	12326 (48.95)	1929 (30.64)	2776 (44.10)	3318 (52.71)	4303 (68.36)	
Female	12855 (51.05)	4367 (69.36)	3519 (55.90)	2977 (47.29)	1992 (31.64)	
Race (%)						0.328
Mexican American	3745 (14.87)	880 (13.98)	926 (14.71)	957 (15.20)	982 (15.60)	
Other Hispanic	2651 (10.53)	647 (10.28)	667 (10.60)	675 (10.72)	662 (10.52)	
Non-Hispanic White	10050 (39.91)	2517 (39.98)	2526 (40.13)	2525 (40.11)	2482 (39.43)	
Non-Hispanic Black	5207 (20.68)	1372 (21.79)	1279 (20.32)	1256 (19.95)	1300 (20.65)	
Others Race	3528 (14.01)	880 (13.98)	897 (14.25)	882 (14.01)	869 (13.80)	
Poverty income rate (%)						0.680
Low (<1)	5556 (22.06)	1431 (22.73)	1406 (22.34)	1375 (21.84)	1344 (21.35)	
Middle (1–4)	13279 (52.73)	3297 (52.37)	3309 (52.57)	3327 (52.85)	3346 (53.15)	
High (>4)	6346 (25.20)	1568 (24.90)	1580 (25.10)	1593 (25.31)	1605 (25.50)	
Education levels (%)						0.241
High school or less	11666 (46.33)	2872 (45.62)	2889 (45.89)	2928 (46.51)	2977 (47.29)	
More than high school	13515 (53.67)	3424 (54.38)	3406 (54.11)	3367 (53.49)	3318 (52.71)	
Marital status (%)						<0.001
Married	12876 (51.13)	3084 (48.98)	3254 (51.69)	3283 (52.15)	3255 (51.71)	
Widowed	1924 (7.64)	550 (8.74)	495 (7.86)	409 (6.50)	470 (7.47)	
Divorced	2770 (11.00)	758 (12.04)	702 (11.15)	652 (10.36)	658 (10.45)	
Separated	864 (3.43)	238 (3.78)	233 (3.70)	202 (3.21)	191 (3.03)	
Living with partner	2081 (8.26)	514 (8.16)	501 (7.96)	537 (8.53)	529 (8.40)	
Never married	4666 (18.53)	1152 (18.30)	1110 (17.63)	1212 (19.25)	1192 (18.94)	
Smoking (%)						<0.001
Yes	11016 (43.75)	2523 (40.07)	2440 (38.76)	2800 (44.48)	3253 (51.68)	
No	14165 (56.25)	3773 (59.93)	3855 (61.24)	3495 (55.52)	3042 (48.32)	
drinking (%)						0.155
Yes	17115 (67.97)	4222 (67.06)	4260 (67.67)	4298 (68.28)	4335 (68.86)	
No	8066 (32.03)	2074 (32.94)	2035 (32.33)	1997 (31.72)	1960 (31.14)	
BMI, kg/m^2^						<0.001
<25	7089 (28.15)	1874 (29.76)	1816 (28.85)	1777 (28.23)	1622 (25.77)	
25-30	8345 (33.14)	1869 (29.69)	2116 (33.61)	2129 (33.82)	2231 (35.44)	
≥30	9747 (38.71)	2553 (40.55)	2363 (37.54)	2389 (37.95)	2442 (38.79)	
CKD (%)						<0.001
Yes	4307 (17.10)	1334 (21.19)	1176 (18.68)	992 (15.76)	805 (12.79)	
No	20874 (82.90)	4962 (78.81)	5119 (81.32)	5303 (84.24)	5490 (87.21)	
Hypertension (%)						0.411
Yes	10551 (41.90)	2692 (42.76)	2634 (41.84)	2602 (41.33)	2623 (41.67)	
No	14630 (58.10)	3604 (57.24)	3661 (58.16)	3693 (58.67)	3672 (58.33)	
DM (%)						0.264
Yes	4822 (19.15)	1250 (19.85)	1202 (19.09)	1162 (18.46)	1208 (19.19)	
No	20359 (80.85)	5046 (80.15)	5093 (80.91)	5133 (81.54)	5087 (80.81)	
CVD (%)						0.203
Yes	2075 (8.24)	542 (8.61)	504 (8.01)	541 (8.59)	488 (7.75)	
No	23106 (91.76)	5754 (91.39)	5791 (91.99)	5754 (91.41)	5807 (92.25)	
Dyslipidemia (%)						0.188
Yes	17691 (70.26)	4373 (69.46)	4404 (69.96)	4434 (70.44)	4480 (71.17)	
No	7490 (29.74)	1923 (30.54)	1891 (30.04)	1861 (29.56)	1815 (28.83)	
Age, years	50.79 ± 14.60	53.67 ± 14.42	51.39 ± 14.56	50.06 ± 14.12	48.02 ± 14.71	<0.001
eGFR, mL/min/1.73 m^2^	90.02 ± 20.79	80.92 ± 19.23	87.45 ± 19.79	92.44 ± 19.72	99.29 ± 19.94	<0.001
ACR, mg/g	46.92 ± 350.85	83.89 ± 578.24	42.52 ± 288.94	33.14 ± 221.43	28.11 ± 153.58	<0.001
BUN, mmol/L	4.99 ± 1.74	5.12 ± 1.73	5.00 ± 1.75	4.94 ± 1.74	4.89 ± 1.71	<0.001
SCr, μmol/L	78.07 ± 14.71	81.82 ± 14.50	79.32 ± 14.55	76.82 ± 14.39	74.33 ± 14.33	<0.001
SUA, μmol/L	324.83 ± 84.56	339.25 ± 83.61	331.32 ± 82.93	320.39 ± 84.72	308.36 ± 83.76	<0.001
HGB, g/L	139.75 ± 11.93	130.93 ± 10.22	137.65 ± 9.85	141.93 ± 10.00	148.48 ± 10.23	<0.001
LYM, × 10^9^/L	2.11 ± 0.85	1.31 ± 0.29	1.77 ± 0.32	2.23 ± 0.41	3.13 ± 0.85	<0.001
PLT, × 10^9^/L	241.51 ± 48.84	278.40 ± 40.79	252.61 ± 39.11	233.35 ± 38.41	201.69 ± 41.68	<0.001
ALB, g/L	41.64 ± 2.84	40.16 ± 2.66	41.30 ± 2.59	42.03 ± 2.61	43.08 ± 2.66	<0.001
ALT, U/L	26.08 ± 17.00	23.31 ± 16.56	24.13 ± 14.42	26.59 ± 16.66	30.30 ± 19.16	<0.001
AST, U/L	25.64 ± 14.13	23.41 ± 13.98	24.50 ± 12.54	26.15 ± 13.72	28.49 ± 15.59	<0.001
LDL-C, mmol/L	2.90 ± 0.92	2.89 ± 0.94	2.91 ± 0.93	2.91 ± 0.92	2.90 ± 0.91	0.569
HDL-C, mmol/L	1.40 ± 0.72	1.41 ± 0.72	1.40 ± 0.71	1.39 ± 0.72	1.38 ± 0.73	0.110
TC, mmol/L	5.00 ± 1.04	4.99 ± 1.05	5.00 ± 1.03	5.01 ± 1.05	5.02 ± 1.03	0.408
TG, mmol/L	1.80 ± 1.04	1.68 ± 1.01	1.72 ± 1.03	1.83 ± 1.04	1.99 ± 1.06	<0.001

HALP score = hemoglobin, albumin, lymphocyte, and platelet score

PIR = poverty-to-income ratio, BMI = body mass index, CKD = chronic kidney disease, DM = diabetes mellitus, CVD = cardiovascular disease, eGFR = estimated glomerular filtration rate, ACR = urine albumin-to-creatinine ratio, BUN = blood urea nitrogen, Scr = serum creatinine, HGB = hemoglobin, LYM = lymphocytes, PLT = platelet, ALB = serum albumin, ALT = alanine transaminase, AST = aspartate transaminase, LDL-C = low-density lipoprotein cholesterol, HDL-C = high-density lipoprotein cholesterol, TC = total cholesterol, TG = triglycerides.

### Relationship between HALP score and CKD, albuminuria, and low-eGFR

#### Relationship between HALP score and CKD.

As shown in Table 2, a higher HALP score was associated with reduced CKD prevalence in all models. Furthermore, when treated as a continuous variable, each additional point in the HALP score was associated with a 3% lower prevalence of CKD (OR = 0.97, 95% CI: 0.94–0.99, *P* = 0.031). Consistent results were observed in quartile-based analyses, with participants in the top quartile having markedly lower odds of CKD than those in the bottom quartile in Model 3 (OR = 0.63, 95% CI: 0.52–0.76, *P* = 0.001).

**Table 2:** Associations between HALP score and CKD, albuminuria, and low-eGFR.

	Crude model (Model 1)^3^		Adjusted model (Model 2)^4^		Adjusted model (Model 3)^5^	
	OR^1^(95%CI^2^)	*P* value	OR (95%CI)	*P* value	OR (95%CI)	*P* value
CKD						
continuous	0.95 (0.92-0.98)	0.002	0.96 (0.93-0.99)	0.016	0.97 (0.94-0.99)	0.031
Q1	Reference		Reference		Reference	
Q2	0.85 (0.75-0.96)	0.010	0.86 (0.77-0.96)	0.035	0.89 (0.80-0.98)	0.048
Q3	0.70 (0.61-0.80)	<0.001	0.73 (0.63-0.84)	<0.001	0.78 (0.66-0.92)	0.004
Q4	0.55 (0.47-0.64)	<0.001	0.58 (0.49-0.68)	<0.001	0.63 (0.52-0.76)	0.001
*P* for trend	<0.001		<0.001		0.008	
Albuminuria						
continuous	0.94 (0.91-0.97)	<0.001	0.95 (0.92-0.98)	0.003	0.97 (0.93-0.99)	0.044
Q1	Reference		Reference		Reference	
Q2	0.82 (0.72-0.93)	0.003	0.85 (0.74-0.97)	0.017	0.94 (0.79-1.09)	0.095
Q3	0.68 (0.59-0.78)	< 0.001	0.71 (0.61-0.82)	< 0.001	0.75 (0.64-0.88)	0.001
Q4	0.52 (0.44-0.61)	< 0.001	0.55 (0.46-0.65)	< 0.001	0.60 (0.49-0.73)	0.001
*P* for trend	< 0.001		<0.001		0.006	
Low-eGFR						
continuous	0.96 (0.93-0.99)	0.015	0.97 (0.94-0.99)	0.021	0.98 (0.95-1.01)	0.065
Q1	Reference		Reference		Reference	
Q2	0.89 (0.81-0.98)	0.020	0.91 (0.82-0.99)	0.042	0.95 (0.85-1.06)	0.089
Q3	0.85 (0.76-0.95)	0.007	0.88 (0.79-0.98)	0.022	0.91 (0.81-1.02)	0.078
Q4	0.78 (0.70-0.87)	0.002	0.81 (0.73-0.90)	0.008	0.88 (0.75-1.01)	0.055
*P* for trend	0.005		0.018		0.062	

^1^OR: Odd ratio.

^2^95% CI: 95% confidence interval.

^3^Model 1: No covariates were adjusted.

^4^Model 2: Adjusted for age, gender, and race.

^5^Model 3: Adjusted for age, gender, race, marital status, education level, PIR, BMI, smoking, drinking, AST, ALT, hypertension, DM, CVD, and dyslipidemia.

HALP score = hemoglobin, albumin, lymphocyte, and platelet score, CKD = chronic kidney disease, eGFR = estimated glomerular filtration rate, PIR = poverty-to-income ratio, BMI = body mass index, AST = aspartate transaminase, ALT = alanine transaminase, DM = diabetes mellitus, CVD = cardiovascular disease.

Smooth curve fitting further indicated a non-linear connection between the HALP score and prevalent CKD (Fig 2A). Specifically, CKD prevalence decreased as the HALP score increased, reaching its lowest point at a HALP score threshold of 52.43 (OR = 0.97, 95% CI: 0.95–0.99, *P* < 0.05). Beyond this threshold, no further significant association with prevalent CKD was observed (OR = 1.00, 95% CI: 0.98–1.01, *P* = 0.786) (Table 3).

**Table 3:** Threshold association analysis of the HALP score with CKD using a two-piecewise linear regression model after adjustment of covariates.

CKD	Adjusted OR^1^ (95%CI^2^)	*P* value
Fitting by the standard linear model	0.97 (0.94-0.99)	0.032
Fitting by the two-piecewise linear model		
HALP score		
Inflection point	52.43	
HALP score < 52.43	0.97 (0.95-0.99)	0.023
HALP score ≥ 52.43	1.00 (0.98-1.01)	0.786
Log likelihood ratio		0.031

^1^OR: Odd ratio

^2^95% CI: 95% confidence interval

Adjusted for age, gender, race, marital status, education level, PIR, BMI, smoking, drinking, AST, ALT, hypertension, DM, CVD, and dyslipidemia.

HALP score = hemoglobin, albumin, lymphocyte, and platelet score, CKD = chronic kidney disease, PIR = poverty-to-income ratio, BMI = body mass index, AST = aspartate transaminase, ALT = alanine transaminase, DM = diabetes mellitus, CVD = cardiovascular disease.

**Fig 2:**
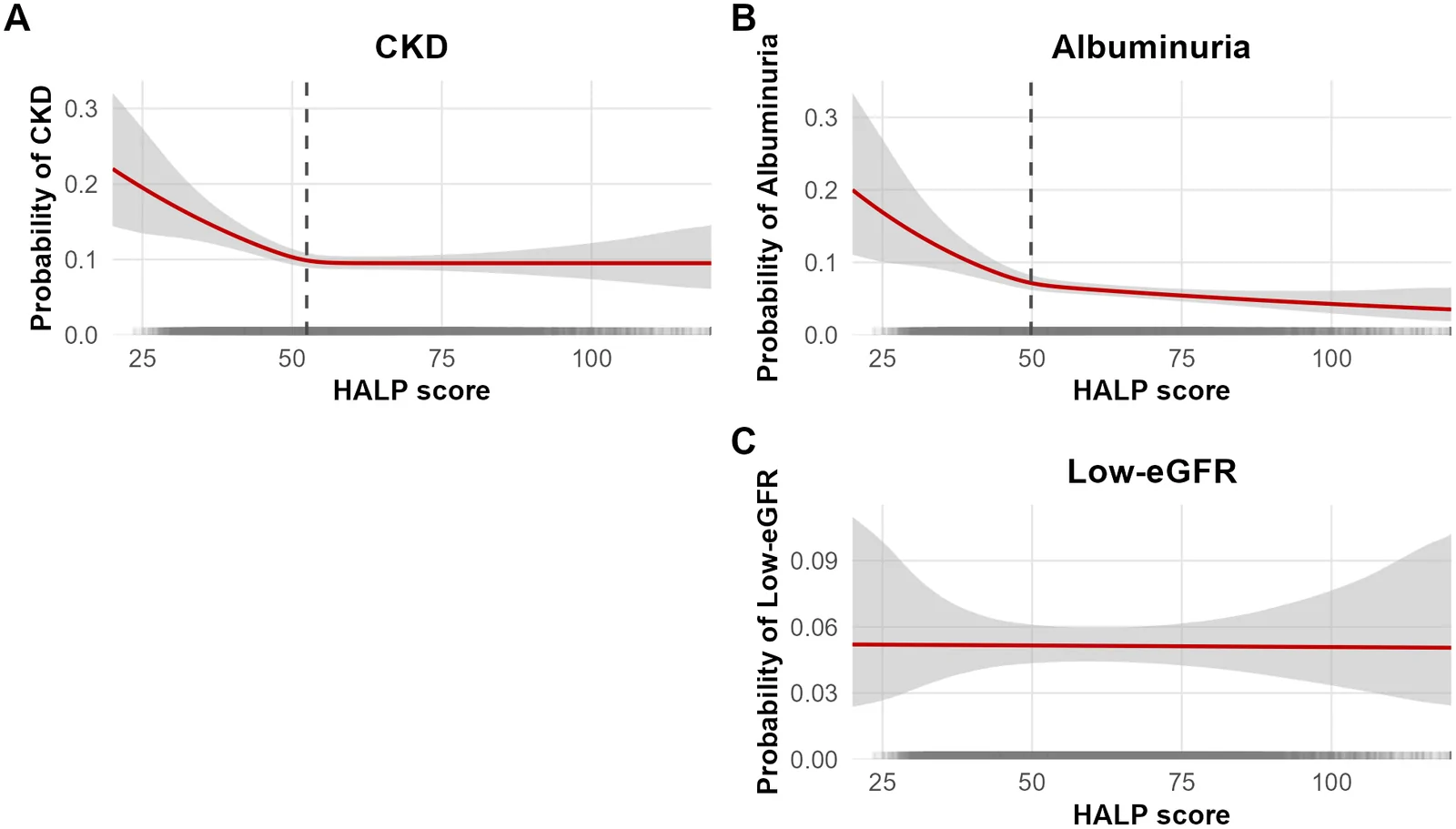
Smooth curve fitting depicting the non-linear associations of the HALP score with CKD (A), albuminuria (B), and low-eGFR (C), assessed using generalized additive models. The solid lines represent the fitted curves, and the shaded areas represent the 95% confidence intervals. A HALP score and CKD; B HALP score and albuminuria; C HALP score and low-eGFR Abbreviations: CKD = chronic kidney disease, eGFR = estimated glomerular filtration rate, HALP score = hemoglobin, albumin, lymphocyte, and platelet score.

#### Relationship between HALP score and albuminuria.

A lower HALP score was similarly associated with a higher prevalence of albuminuria (OR = 0.97, 95% CI: 0.93–0.99, *P* = 0.044). This association persisted when the HALP score was categorized into quartiles, with participants in lower quartiles showing a higher prevalence of albuminuria compared with those in higher quartiles (trend test, *P* < 0.05) (Table 2).

Smooth curve fitting revealed a non-linear, logarithmic-like relationship between the HALP score and albuminuria, with an inflection point of 49.82 (Fig 2B). Prior to the threshold, every single-point rise in HALP score was associated with a 4% lower prevalence of albuminuria (OR = 0.96, 95% CI: 0.94–0.98, *P* < 0.05), whereas above the threshold, the association was not significant (OR = 0.99, 95% CI: 0.97–1.01, *P* = 0.352) (Table 4).

**Table 4:** Threshold association analysis of the HALP score with albuminuria using a two-piecewise linear regression model after adjustment of covariates.

Albuminuria	Adjusted OR^1^ (95%CI^2^)	*P* value
Fitting by the standard linear model	0.96 (0.93-0.99)	0.018
Fitting by the two-piecewise linear model		
HALP score		
Inflection point	49.82	
HALP score < 49.82	0.96 (0.94-0.98)	0.007
HALP score ≥ 49.82	0.99 (0.97-1.01)	0.352
Log likelihood ratio		0.025

^1^OR: Odd ratio

^2^95% CI: 95% confidence interval

Adjusted for age, gender, race, marital status, education level, PIR, BMI, smoking, drinking, AST, ALT, hypertension, DM, CVD, and dyslipidemia.

HALP score = hemoglobin, albumin, lymphocyte, and platelet score, PIR = poverty-to-income ratio, BMI = body mass index, AST = aspartate transaminase, ALT = alanine transaminase, DM = diabetes mellitus, CVD = cardiovascular disease.

#### Relationship between HALP score and low-eGFR.

In Model 1, the HALP score showed a significant inverse association with low-eGFR (OR = 0.96, 95% CI: 0.93–0.99, *P* < 0.05), and this association persisted in Model 2 (OR = 0.97, 95% CI: 0.94–0.99, *P* < 0.05). However, in Model 3, the association lost statistical significance (OR = 0.98, 95% CI: 0.95–1.01, *P* = 0.065) (Table 2). Furthermore, smooth curve fitting did not reveal a significant non-linear association between the HALP score and low-eGFR (Fig 2C).

### Subgroup analysis

Subgroup analyses stratified by age, gender, hypertension, DM, and BMI were performed. Interaction analyses indicated that none of the stratification variables significantly modified the association between the HALP score and CKD (all *P* > 0.05). Similarly, the associations of the HALP score with albuminuria and low-eGFR remained stable across all evaluated subgroups (Fig 3).

**Fig 3:**
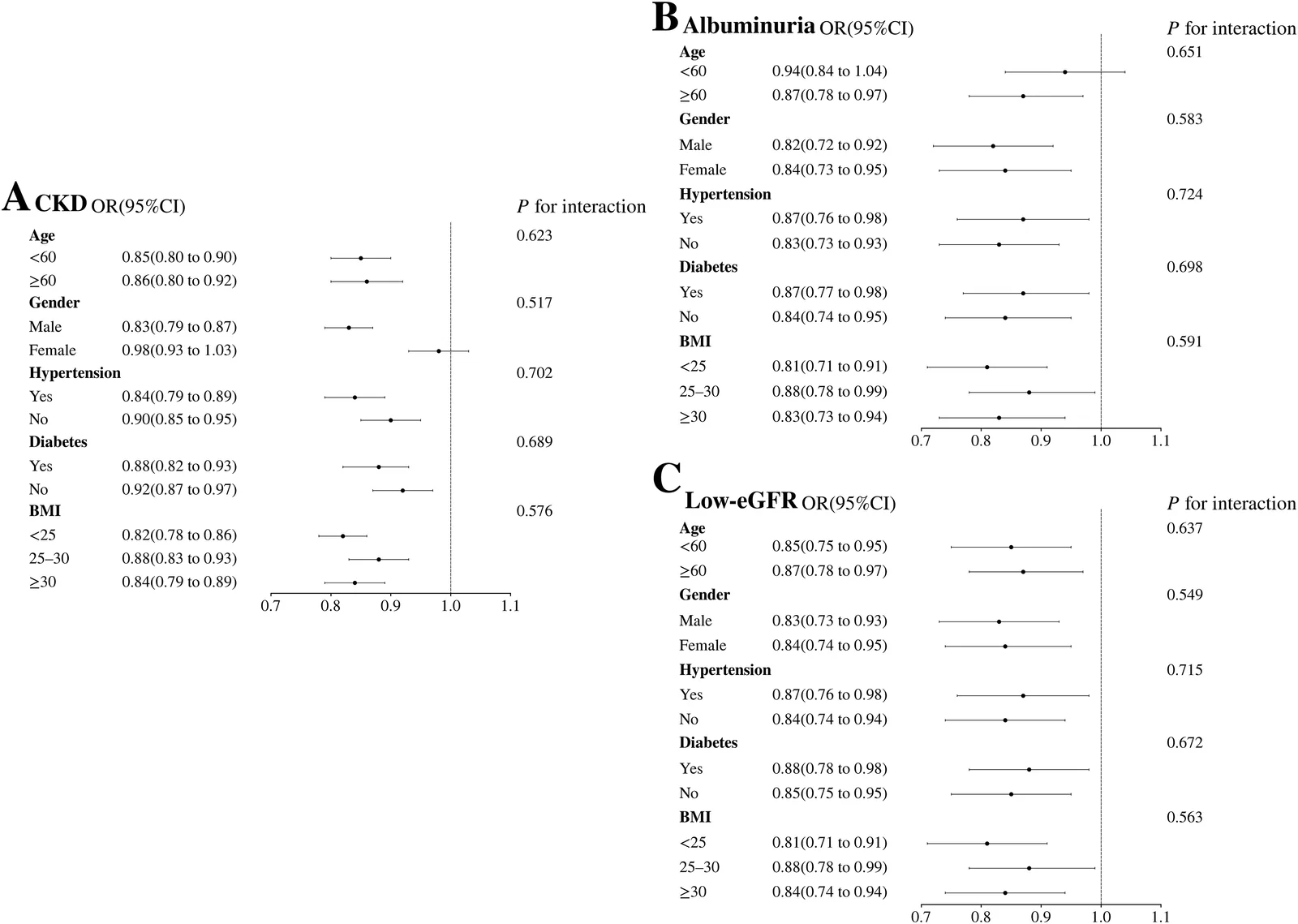
Subgroup analysis of the associations between the HALP score and CKD, albuminuria, and low-eGFR. A HALP score and CKD; B HALP score and albuminuria; C HALP score and low-eGFR. Abbreviations: CKD = chronic kidney disease, eGFR = estimated glomerular filtration rate, HALP score = hemoglobin, albumin, lymphocyte, and platelet score.

### Sensitivity analysis

To verify robustness of our findings, we performed sensitivity analyses by excluding participants with extreme HALP scores (lowest and highest 5%). The fully adjusted Model 3 was reapplied, and the results were consistent with the main analyses, confirming the stability of the inverse association between the HALP score and CKD (OR = 0.97, 95% CI: 0.94–0.99, *P* < 0.05) (Table 5).

**Table 5:** Sensitivity analysis after excluding extreme 5% of HALP score.

	Crude model (Model 1)^3^		Adjusted model (Model 2)^4^		Adjusted model (Model 3)^5^	
	OR^1^(95%CI^2^)	*P* value	OR (95%CI)	*P* value	OR (95%CI)	*P* value
CKD						
continuous	0.95 (0.92-0.98)	0.003	0.96 (0.93-0.99)	0.018	0.97 (0.94-0.99)	0.041
Q1	Reference		Reference		Reference	
Q2	0.86 (0.76-0.97)	0.012	0.87 (0.77-0.98)	0.038	0.93 (0.81-1.05)	0.072
Q3	0.71 (0.62-0.81)	<0.001	0.74(0.63-0.85)	<0.001	0.80 (0.67-0.93)	0.005
Q4	0.56 (0.48-0.65)	<0.001	0.59 (0.49-0.69)	<0.001	0.65 (0.53-0.78)	0.002
*P* for trend		<0.001		<0.001		0.008

^1^OR: Odd ratio

^2^95% CI: 95% confidence interval

^3^Model 1: No covariates were adjusted

^4^Model 2: Adjusted for age, gender, and race

^5^Model 3: Adjusted for age, gender, race, marital status, education level, PIR, BMI, smoking, drinking, AST, ALT, hypertension, DM, CVD, and dyslipidemia.

HALP score = hemoglobin, albumin, lymphocyte, and platelet score, CKD = chronic kidney disease, PIR = poverty-to-income ratio, BMI = body mass index, AST = aspartate transaminase, ALT = alanine transaminase, DM = diabetes mellitus, CVD = cardiovascular disease.

### ROC analysis

ROC curve analyses were performed to compare the discriminative ability of the HALP score with other biomarkers (PNI, PLR, LMR, and SII) for CKD, albuminuria, and low-eGFR (Fig 4). The results revealed that HALP scores had higher AUC values than other biomarkers, with significant differences for CKD and albuminuria (all *P* < 0.05, Table 6). For low-eGFR, the HALP–LMR difference was not significant (*P* = 0.85). These findings suggest that the HALP score offers a modest discriminative advantage for CKD and albuminuria compared with other inflammation- and nutrition-related biomarkers.

**Table 6:** Comparison of AUC values between HALP score and other markers for discriminating CKD, albuminuria, and low-eGFR.

Test	AUC^1^	95% CI^2^low	95% CI upp	Best threshold	Specificity	Sensitivity	DeLong *P* value
CKD							
HALP score	0.70	0.68	0.72	52.34	0.65	0.70	Reference
PNI	0.66	0.64	0.68	42.15	0.58	0.62	<0.001
PLR	0.63	0.61	0.65	155.78	0.55	0.58	<0.001
LMR	0.67	0.65	0.69	3.89	0.62	0.66	<0.001
SII	0.62	0.60	0.64	610.23	0.52	0.56	<0.001
Albuminuria							
HALP score	0.70	0.67	0.71	51.23	0.50	0.75	Reference
PNI	0.65	0.63	0.67	41.34	0.46	0.72	<0.001
PLR	0.64	0.62	0.66	154.89	0.58	0.56	<0.001
LMR	0.66	0.64	0.68	3.78	0.42	0.70	<0.001
SII	0.63	0.61	0.65	605.34	0.38	0.68	<0.001
Low-eGFR							
HALP score	0.71	0.69	0.73	53.45	0.66	0.52	Reference
PNI	0.67	0.65	0.69	43.21	0.52	0.63	<0.001
PLR	0.65	0.63	0.67	156.78	0.59	0.55	<0.001
LMR	0.68	0.66	0.70	3.90	0.61	0.54	0.85
SII	0.64	0.62	0.66	615.45	0.59	0.55	<0.001

^1^AUC: area under the curve

^2^95% CI: 95% confidence interval

HALP score = hemoglobin, albumin, lymphocyte, and platelet score, CKD = chronic kidney disease, eGFR = estimated glomerular filtration rate, PNI = prognostic nutritional index, PLR = platelet-to-lymphocyte ratio LMR = lymphocyte-to-monocyte ratio, SII = systemic immune-inflammation index.

**Fig 4:**
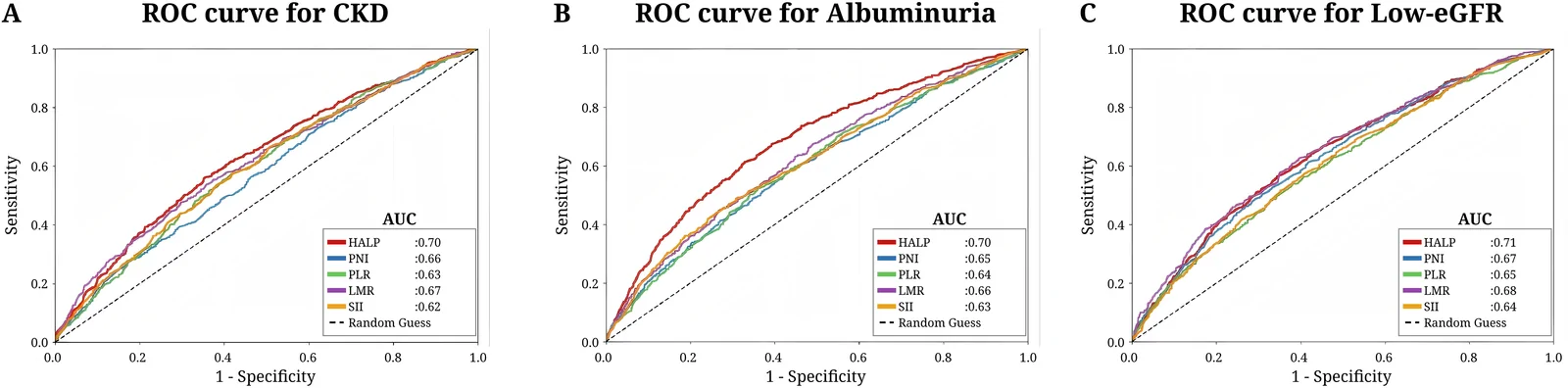
ROC curves and AUC values of the five markers (HALP score, PNI, PLR, LMR, and SII) for CKD, albuminuria and low-eGFR. A Five markers were assessed to discriminate CKD; B Five markers were assessed to discriminate albuminuria; C Five markers were assessed to discriminate low-eGFR. Abbreviations: ROC = receiver operating characteristic, AUC = area under the curve, CKD = chronic kidney disease, eGFR = estimated glomerular filtration rate, HALP score = hemoglobin, albumin, lymphocyte, and platelet score, PNI = prognostic nutritional index, LMR = lymphocyte-to-monocyte ratio, PLR = platelet-to-lymphocyte ratio, SII = systemic immune-inflammation index.

### Findings from the 5/6 nephrectomy rat model

A reliable CKD rat model was successfully established. In the CON group, H&E staining showed normal glomerular morphology with regularly arranged capillary loops, and no obvious mesangial proliferation or matrix accumulation was observed. Renal tubules were regularly organized with intact epithelial cells and clear lumens, and no inflammatory infiltration or interstitial fibrosis was observed. By contrast, in the model group, glomeruli exhibited mesangial cell proliferation and increased matrix deposition, accompanied by disordered capillary loops, thickened Bowman’s capsule, and collapsed capillary tufts. Renal tubules were disordered with irregular luminal dilation, along with varying degrees of tubular epithelial degeneration, necrosis, and shedding. Marked inflammatory cell infiltration was also detected in the renal interstitium. Masson trichrome staining demonstrated widespread deposition of blue-stained collagen fibers in the model group, including glomerular mesangial areas, peri-Bowman’s capsule regions, and the renal interstitium, indicating interstitial fibrosis (Fig 5).

**Fig 5:**
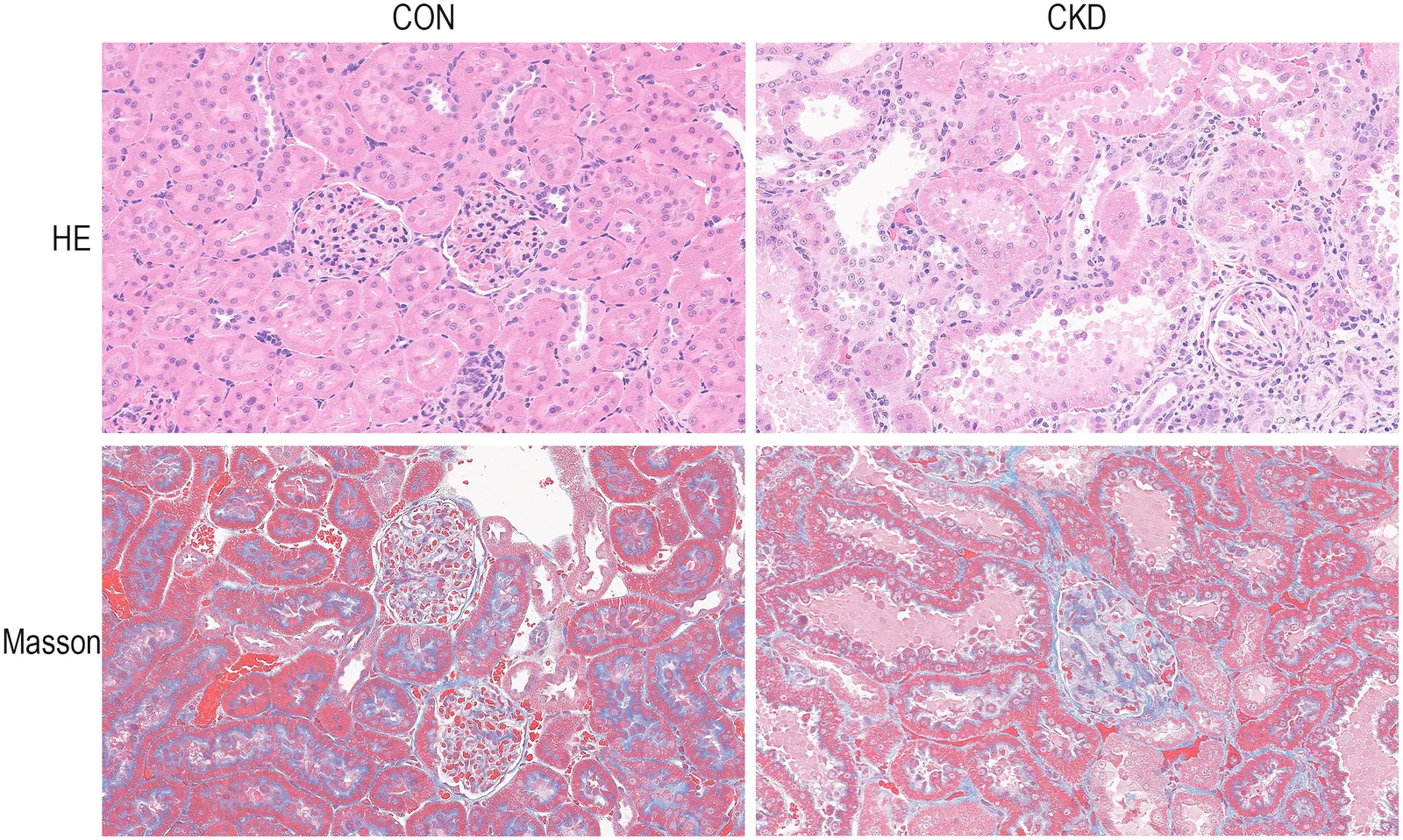
Representative HE and Masson staining of rat renal tissues. Scale bars: 50μm.

Compared with the CON group, CKD model rats exhibited significantly elevated serum BUN, Scr, and UACR levels (all *P* < 0.05, Table 7, Fig 6). Meanwhile, CKD rats presented markedly decreased calculated rat HALP values, accompanied by reduced Hb, ALB, and LYM levels and increased PLT counts (all *P* < 0.05, Table 7, Fig 6).

**Table 7:** Comparison of HB, ALB, LYM, PLT, calculated HALP values, BUN, Scr, and UACR levels in rat models (mean ± SEM, n = 10).

group	HB(g/L)	ALB(g/L)	LYM(10^9^/L)	PLT(10^9^/L)	HALP value	BUN (mmol/L)	Scr (umol/L)	UACR (mg/g)
CON	154.40±2.39	35.83±0.25	10.36±0.56	1470.40±41.42	38.97±1.90	6.04± 0.36	34.76± 1.54	114.28± 10.05
CKD	144.50±3.36	34.24±0.64	8.51±0.63	1609.00±47.80	26.37±2.21	9.33± 0.25	59.24± 2.25	272.26± 35.20
*t*	2.402	2.325	2.207	-2.191	4.322	-7.503	-8.971	-4.316
*P*	0.027	0.039	0.041	0.042	<0.001	<0.001	<0.001	0.001

HB = Hemoglobin, ALB = Albumin, LYM = lymphocytes, PLT = Platelet, HALP value = Hemoglobin, Albumin, Lymphocyte and Platelet value, BUN = Blood Urea Nitrogen, Scr = serum creatinine, UACR = Urinary Albumin-to-Creatinine Ratio

**Fig 6:**
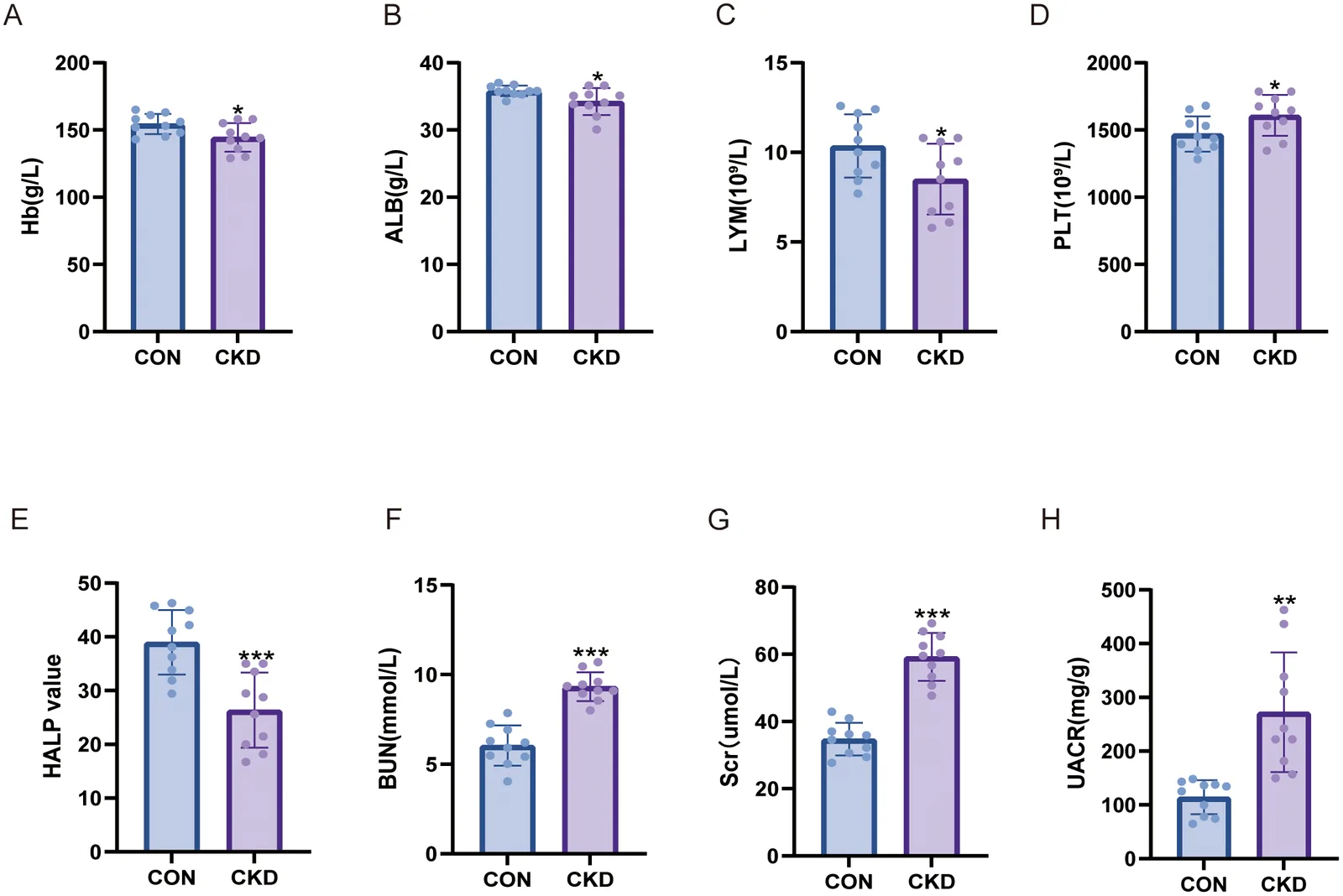
Comparison of hematological and renal function-related parameters between the control CON and CKD groups (A) Hb; (B) ALB; (C) LYM; (D) PLT; (E) HALP value; (F) BUN; (G) Scr; (H) UACR. Data are presented as mean ± SEM; *P < 0.05, **P < 0.01, ***P < 0.001 vs. the CON group. HB = Hemoglobin, ALB = Albumin, LYM = lymphocytes, PLT = Platelet, HALP value = Hemoglobin, Albumin, Lymphocyte and Platelet value, BUN = Blood Urea Nitrogen, Scr = serum creatinine, UACR = Urinary Albumin-to-Creatinine Ratio.

### Exploratory correlations of calculated HALP values with renal function indicators

Pearson correlation analysis showed that calculated HALP values were inversely correlated with BUN (r = −0.636, 95% CI: −0.875 to −0.341, *P* = 0.003), Scr (r = −0.639, 95% CI: −0.853 to −0.368, *P* = 0.002), and UACR (r = −0.771, 95% CI: −0.891 to −0.543, *P* < 0.001) in rats (Table 8).

**Table 8:** Pearson correlations between calculated HALP value and renal function indicators in rats (n = 20).

Parameter	*r*	*95% CI*	*P*
BUN	−0.636	(−0.875, −0.341)	0.003
Scr	−0.639	(−0.853, −0.368)	0.002
UACR	−0.771	(−0.891, −0.543)	<0.001

HALP value = Hemoglobin, Albumin, Lymphocyte and Platelet value, BUN = Blood Urea Nitrogen, Scr = serum creatinine, UACR = Urinary Albumin-to-Creatinine Ratio

## Discussion

Our findings extend previous evidence linking HALP with renal disease to the general adult population and provide complementary experimental support from a CKD rat model. CKD is often asymptomatic in its early stages and may progress before detection [15]. Chronic inflammation and nutritional disturbances are closely intertwined in CKD and may contribute to renal injury and disease progression [16,17]. The HALP score, an integrated composite metric reflecting both inflammatory and nutritional status, has been linked to a variety of conditions, including overactive bladder syndrome [18], coronary heart disease [19], and psoriasis [20]. Specifically in the renal context, reduced HALP scores correlate with advanced tubular atrophy and interstitial fibrosis in IgAN [21]. However, prior studies have largely focused on specific renal disease subtypes, such as DN, IgAN, lupus nephritis, and dialysis populations, with relatively few investigations focusing on the general adult population using large nationally representative datasets. In the present NHANES analysis, we observed a robust inverse association between the HALP score and prevalent CKD, and notably identified a non-linear threshold pattern that warrants further discussion.

The HALP score threshold identified in our analysis (52.43) is exploratory before clinical use. Notably, similar non-linear, threshold-type associations have been reported for HALP in other disease contexts. Ding et al. [22] reported an L-shaped relationship between the HALP score and diabetic retinopathy, with risk declining sharply until plateauing above a threshold of 42.9. Similarly, a threshold of 44.98 was reported for diabetic foot ulcer risk [23]. These consistent threshold patterns across different diseases suggest that the HALP score may reflect common underlying pathophysiological mechanisms. The potential utility of the identified threshold as complementary information alongside established renal measures should therefore be evaluated in future prospective studies.

The differential associations of the HALP score with albuminuria and low-eGFR warrant careful interpretation. One possible explanation is that albuminuria may reflect glomerular injury at an earlier stage than reduced eGFR [24]. However, equally plausible explanations are structural overlap and reverse causation. Serum albumin may decline with albuminuria due to urinary protein loss, while hemoglobin may decline as renal function deteriorates because of reduced erythropoietin production. To assess whether the observed association was confined to these renally related components, we performed a component-resolved sensitivity analysis, modeling each HALP component separately in fully adjusted Model 3 (S1 Table). Lymphocytes and platelets remained associated with CKD and albuminuria, suggesting that the observed HALP association was not confined to hemoglobin and albumin. Nevertheless, reverse causation cannot be excluded given the cross-sectional design. Subgroup analyses further showed that the association between the HALP score and CKD remained consistent across diverse subgroups. Given the high CKD risk among individuals with hypertension, diabetes [25], or advanced age [26], the clinical relevance of HALP in these high-risk groups warrants further investigation. Taken together with the findings from the component-resolved analyses, these results support the potential role of HALP as a complementary marker for CKD assessment.

The modest AUC advantage of the HALP score compared with PNI, PLR, LMR, and SII may reflect its integration of multiple potentially CKD-related pathophysiological pathways, although component overlap may also contribute. Based on existing literature, decreased hemoglobin has been associated with renal hypoxia and activation of the sympathetic and renin-angiotensin-aldosterone systems, potentially aggravating kidney injury [27]. Second, serum albumin exhibits antioxidant, anti-inflammatory, and antiplatelet aggregation properties, which may contribute to renal protection [28]. Third, lymphocytes and their mediators are thought to help mitigate renal inflammation and fibrosis, and lymphopenia has been linked to immune dysregulation in CKD [29]. Fourth, activated platelets may promote CKD progression through microthrombosis formation, release of inflammatory mediators, and profibrotic signaling [30]. Collectively, these complementary pathways may help explain the observed association between HALP and CKD.

Furthermore, we incorporated a 5/6 nephrectomy rat model. CKD rats exhibited typical renal dysfunction and characteristic pathological damage compared with controls, confirming successful model establishment [31]. Importantly, CKD rats showed significantly lower calculated HALP values, accompanied by reduced Hb, ALB, and LYM levels and elevated PLT counts. Pearson correlation analysis further revealed significant negative correlations between the calculated HALP values and Scr, BUN, and UACR. Although albumin was measured by BCP in NHANES and BCG in rats, making absolute HALP values not directly comparable across species, these animal findings further support the biological plausibility of the observed HALP–CKD association [32].

Building on the above findings, several strengths of this study deserve further emphasis. First, this work systematically evaluates the HALP-CKD relationship in a large, nationally representative general population, extending prior work that has been largely confined to specific renal disease subtypes (e.g., DN, IgAN, lupus nephritis, dialysis). Second, this exploratory threshold may provide a quantitative reference for future studies assessing its potential clinical utility in populations with a higher prevalence of CKD, including individuals with hypertension, diabetes, or older age. Third, ROC comparisons demonstrated that the HALP score showed higher AUCs than several established inflammation-nutrition indices (PNI, SII, LMR, PLR) for CKD and albuminuria, supporting its potential discriminative value. Most importantly, the 5/6 nephrectomy rat model provided complementary experimental support for the HALP–CKD association, as CKD rats exhibited lower calculated HALP values alongside characteristic component changes. These findings further support the biological plausibility of this association.

Several limitations of this study should be acknowledged. First, the cross-sectional observational design of the NHANES dataset precludes causal inference regarding the association between the HALP score and CKD. In addition, the single-visit eGFR/ACR-based CKD definition cannot confirm the KDIGO ≥3-month chronicity criterion; transient abnormalities, including albuminuria, may be included, potentially overestimating CKD prevalence, and the study design precludes assessment of CKD incidence or progression. Second, reverse causation and partial structural overlap remain important concerns because hemoglobin and albumin may be altered by renal dysfunction, with albumin additionally affected by urinary protein loss, particularly in relation to albuminuria. Although the component-resolved analysis (S1 Table) suggests that the observed HALP association is not confined to these two components, prospective data are needed to establish temporal direction. Third, despite comprehensive adjustment for known confounders, residual confounding by unmeasured factors cannot be fully eliminated. Fourth, the proposed links between the HALP score and CKD—via inflammation, malnutrition, and platelet activation—remain speculative, because relevant mechanistic biomarkers and molecular pathways were not examined in this study. These hypotheses require validation in future experiments. Finally, although the animal data provide corroborative support for the directionality of the HALP components, causality remains unestablished. Longitudinal studies and interventional trials targeting nutritional and inflammatory pathways are needed to assess whether such interventions can reduce CKD incidence or slow progression.

## Conclusion

Our integrated population and animal data support the HALP score as a simple biomarker associated with prevalent CKD. Further longitudinal and interventional studies are warranted to establish its predictive utility and to elucidate the underlying mechanisms.

## Supporting information

S1 Table Component-resolved sensitivity analysis of HALP components in relation to CKD, albuminuria, and low eGFR. (DOCX)

S2 Table Sensitivity analysis using the 2021 race-free CKD-EPI equation. (DOCX)

S1 Fig Restricted cubic spline analysis of the association between HALP and CKD. (TIFF)

S1 File Calculation formulas of other inflammatory-nutritional markers. (DOC)
