# Supplementary material for: Association between the hemoglobin albumin lymphocyte and platelet score and chronic kidney disease: Insights from patient data and animal models
Source: PLoS One. 2026 Oct 14;21(10):e0353085. doi: 10.1371/journal.pone.0353085 (PMC13667822; doi:10.1371/journal.pone.0353085)
Supplement: Supplementary file 1 [file pone.0353085.s001.docx]

| Exposure variable | CKD  OR ( 95% CI) | *P* value | Albuminuria  OR (95% CI) | *P* value | Low-eGFR  OR (95% CI) | *P* value |
| --- | --- | --- | --- | --- | --- | --- |
| Hemoglobin | 0.94 (0.89-0.99) | 0.028 | 0.93 (0.88-0.99) | 0.018 | 0.97(0.91-1.03) | 0.321 |
| Albumin | 0.88 (0.82-0.94) | <0.001 | 0.85 (0.79-0.92) | <0.001 | 0.95(0.88-1.03) | 0.218 |
| Lymphocyte count | 0.95 (0.91-0.99) | 0.032 | 0.94 (0.89-0.99) | 0.021 | 0.98(0.92-1.04) | 0.512 |
| Platelet count | 1.05 (1.01-1.09) | 0.019 | 1.06 (1.01-1.11) | 0.014 | 1.01(0.96-1.07) | 0.681 |

**S1 Table. Component-resolved sensitivity analysis of HALP components in relation to CKD, albuminuria, and low-eGFR**

Note: Each HALP component was modeled separately as the exposure variable in fully adjusted Model 3. Model 3 was adjusted for age, gender, race, marital status, education level, poverty-to-income ratio (PIR), body mass index (BMI), smoking, drinking, aspartate transaminase (AST), alanine transaminase (ALT), hypertension, diabetes mellitus (DM), cardiovascular disease (CVD), and dyslipidemia. OR, odds ratio; CI, confidence interval; CKD, chronic kidney disease; eGFR, estimated glomerular filtration rate; HALP, hemoglobin, albumin, lymphocyte, and platelet score.
