# Supplementary material for: Association between the hemoglobin albumin lymphocyte and platelet score and chronic kidney disease: Insights from patient data and animal models
Source: PLoS One. 2026 Oct 14;21(10):e0353085. doi: 10.1371/journal.pone.0353085 (PMC13667822; doi:10.1371/journal.pone.0353085)
Supplement: Supplementary file 2 [file pone.0353085.s002.docx]

| Outcome | Exposure | Model 3 OR (95% CI) | *P* |
| --- | --- | --- | --- |
| CKD | HALP continuous | 0.97 (0.95-0.99) | 0.006 |
|  | Q1 | Reference |  |
|  | Q2 | 0.90 (0.82-0.99) | 0.034 |
|  | Q3 | 0.79 (0.70-0.90) | <0.001 |
|  | Q4 | 0.65 (0.56-0.76) | <0.001 |
|  | *P* for trend |  | <0.001 |
| Low-eGFR | HALP continuous | 0.98 (0.96-1.01) | 0.121 |
|  | Q1 | Reference |  |
|  | Q2 | 0.96 (0.86-1.07) | 0.442 |
|  | Q3 | 0.92 (0.81-1.04) | 0.178 |
|  | Q4 | 0.87 (0.75-1.02) | 0.081 |
|  | *P* for trend |  | 0.094 |

**S2 Table. Sensitivity analysis using the 2021 race-free CKD-EPI equation.**

Note: eGFR was recalculated using the 2021 race-free CKD-EPI creatinine equation. Low-eGFR was defined as eGFR < 60 mL/min/1.73 m², and CKD as albuminuria (ACR ≥ 30 mg/g) or low-eGFR. Estimates were derived from fully adjusted Model 3, including age, gender, race, marital status, education level, PIR, BMI, smoking, drinking, AST, ALT, hypertension, DM, CVD, and dyslipidemia.
