# Supplementary material for: Association between the hemoglobin albumin lymphocyte and platelet score and chronic kidney disease: Insights from patient data and animal models
Source: PLoS One. 2026 Oct 14;21(10):e0353085. doi: 10.1371/journal.pone.0353085 (PMC13667822; doi:10.1371/journal.pone.0353085)
Supplement: Supplementary file 3 [file pone.0353085.s004.doc]

**The calculation formulas of other inflammatory-nutritional markers**

We also compared the predictive power of the HALP score with other markers such as platelet-to-lymphocyte ratio (PLR), lymphocyte-to-monocyte ratio (LMR), systemic immune-inflammation index (SII) and prognostic nutritional index (PNI). The formulas for these markers are as follows: PLR = [platelet count (10⁹/L) / lymphocyte count (10⁹/L)], LMR = [lymphocyte count(10⁹/L) / monocyte count(10⁹/L)], PNI = [serum albumin (g/L) +5 × lymphocyte count(10⁹/L)], and SII = [platelet count(10⁹/L) × neutrophil count(10⁹/L) / lymphocyte count(10⁹/L)].^[1-3]^

**References**

[1]	Gong P, Liu Y, Gong Y, et al. The association of neutrophil to lymphocyte ratio, platelet to lymphocyte ratio, and lymphocyte to monocyte ratio with post-thrombolysis early neurological outcomes in patients with acute ischemic stroke. J Neuroinflammation. 2021;18:51.

[2]	Guo L, Zhao P, Zhu Z. Higher Dietary Inflammatory Index and Systemic Immune-Inflammation Index Score are Associated With Higher Risk of Chronic Kidney Disease: Analysis of the National Health and Nutrition Examination Survey From 1999 to 2018. J Ren Nutr. 2025;35:300-310.

[3]	Yu J H, Chen Y, Yin M G. Association between the prognostic nutritional index (PNI) and all-cause mortality in patients with chronic kidney disease. Ren Fail. 2023;45:2264393.
